# INCIDENCE AND EPIDEMIOLOGY OF ADHESIVE CAPSULITIS DURING THE COVID-19 PANDEMIC

**DOI:** 10.1590/1413-785220233101e261132

**Published:** 2023-02-20

**Authors:** DANILO PASSARO PIRES DE MELLO, JYOTIS NATACHA BRITO CORBIN, LETÍCIA SAKA HOLANDA, LUCIANO PASCARELLI, EDUARDO MISAO NISHIMURA, THIAGO BERNARDO CARVALHO DE ALMEIDA

**Affiliations:** 1 Rede D’Or São Luiz, Hospital IFOR, Shoulder and Elbow Surgery Service, São Bernardo do Campo, SP, Brazil.

**Keywords:** Joint Capsule, Pathology, Shoulder, Bursitis, SARS-CoV-2, COVID-19, Cápsula Articular, Patologia, Ombro, Bursite, SARS-CoV-2, COVID-19

## Abstract

**Objective:**

To evaluate a possible increase of adhesive capsulitis incidence during the COVID-19 pandemic.

**Methods:**

A total of 1,983 patients with shoulder disorders were retrospectively analyzed regarding gender, age, development of adhesive capsulitis and comorbidities (systemic arterial hypertension, diabetes mellitus, dyslipidemia, hypothyroidism, hyperthyroidism, depression, and anxiety) in two different periods: from March 2019 to February 2020 and from March 2020 to February 2021. Descriptive and quantitative variables were statistically analyzed. The program used for the calculations was SPSS 17.0 for Windows.

**Results:**

During the pandemic, there was a 2.41-fold increase (p < 0.001) in cases of adhesive capsulitis (compared to the previous year). Patients with depression and anxiety had a significantly increased risk by 8.8 (p < 0.001) and 14 (p < 0.001) times, respectively, of developing frozen shoulder (regarding the two periods studied).

**Conclusion:**

A significant increase in the incidence of frozen shoulder was observed after the onset of the COVID-19 pandemic in addition to a simultaneous increase of psychosomatic disorders. Prospective studies would help to ratify the idea contained in this research. ***Level of Evidence III, Observational Cross-Sectional Study.***

## RESUMO

**Objetivo:**

Avaliar se houve aumento da incidência de capsulite adesiva durante a pandemia de COVID-19.

**Métodos:**

Foram analisados, retrospectivamente, 1.983 pacientes com desordens do ombro quanto a sexo, idade, desenvolvimento de capsulite adesiva e comorbidades (hipertensão arterial sistêmica, diabetes mellitus, dislipidemia, hipo/hipertireoidismo, depressão e ansiedade) em dois períodos distintos: de março de 2019 a fevereiro de 2020 e de março de 2020 a fevereiro de 2021. Procedeu-se à análise estatística das variáveis descritivas e quantitativas, utilizando o software SPSS 17.0 for Windows para os cálculos.

**Resultados:**

Durante a pandemia, houve aumento de 2,41 vezes (p < 0,001) de casos de capsulite adesiva em relação ao ano anterior. Considerando os períodos estudados, pacientes com depressão e ansiedade apresentaram um risco significativamente aumentado em 8,8 (p < 0,001) e 14 (p < 0,001) vezes, respectivamente, de desenvolver a patologia em questão.

**Conclusão:**

Observou-se um aumento significativo da incidência de ombro congelado após o início da pandemia de COVID-19, além de sua relação com distúrbios psicossomáticos. São necessários estudos prospectivos futuros para ratificar a ideia contida nesta pesquisa. **Nível de Evidência III, Estudo Transversal Observacional.**

## INTRODUCTION

Adhesive capsulitis (AC), or frozen shoulder, is a pathology characterized by progressive pain of spontaneous onset in the shoulder associated with a loss of passive and active movement of the joint.^1^ Such restriction is secondary to inflammation of the joint capsule with consequent thickening and adherence of this structure to itself or to the anatomical neck of humerus.^2^

Although it is often referred to as being anterior,^3^ it may radiate to the anterolateral aspect of the arm and generate discomfort in the region of the deltoid muscle insertion.^1^ ^),(^ ^3^ ^)-(^ ^5^ It sometimes significantly interferes with shoulder functionality and the patient’s quality of sleep.^1^

Such comorbidity has an incidence of approximately 2-5% in the general population,^1^ ^),(^ ^4^ occurring mainly in females aged between 40 and 60 years.^1^ ^),(^ ^4^ ^),(^ ^5^

Its diagnosis is clinical, and the disease can be classified in primary (the frozen shoulder itself) or secondary forms. The latter is subdivided into intrinsic causes (related to shoulder pathologies, such as rotator cuff injuries), extrinsic causes (comorbidities that do not occur in the shoulder but may have a contiguous relation with it, such as Pancoast tumor), or systemic causes (associated to systemic disorders, such as diabetes or thyroid diseases).^5^ ^)-(^ ^9^

In most cases, due to the self-limited nature of the disease, non-surgical treatment is effective. Therapeutic options include physical therapy, the use of symptomatic drugs and intra-articular corticosteroid injections, capsular hydrodilatation, manipulation under anesthesia, and even surgery in refractory cases.^8^

The purpose of this study is to compare the incidence of adhesive capsulitis before and during the pandemic in patients subjected to outpatient care in our department, as well as to evaluate their epidemiological profile.

## MATERIALS AND METHODS

This is an observational retrospective cohort study conducted at an orthopedic hospital located in the municipality of São Bernardo do Campo/SP. This study was approved by the Research Ethics Committee under protocol number 53400321.6.0000.5373.

We used our database to access the medical records of patients subjected to consultations performed at the shoulder and elbow outpatient clinics of our institution in two distinct and consecutive periods: from March 2019 to February 2020 and from March 2020 to February 2021.

During these periods, 5,325 consultations were performed, and the total number of patients was 2,531.

Of these patients, 548 (21.6%) had complaints not related to the shoulder and were excluded from the study.

The remaining 1,983 (79.4%) patients were evaluated for gender, age, development of adhesive capsulitis, and the presence of the following comorbidities: systemic arterial hypertension, diabetes mellitus, dyslipidemia, hypothyroidism, hyperthyroidism, depression, and anxiety.

For frozen shoulder to be considered as adhesive capsulitis, the patient could not present an upper extremity fracture in previous visits, as well as upper extremity surgeries for at least one year.

Since this study aims to analyze the incidence of frozen shoulder in the studied periods, the patients whose medical records contained reports of adhesive capsulitis in consultations before those periods were not considered as having the disease.

### Statistical analysis

Initially, all variables were analyzed descriptively. For quantitative variables, this analysis was performed by observing the minimum and maximum values and the calculation of means, standard deviations, and median. For the qualitative variables, absolute and relative frequencies were estimated.

The student’s *t*-test was used to compare the means of two groups.^10^ The chi-square test^10^ or Fisher’s exact test were used to evaluate homogeneity of proportions.^10^ The program used for calculations was SPSS 17.0 for Windows. The significance level used for the tests was 5%.

## RESULTS

Of the 1983 patients with shoulder-related complaints, 971 (49.0%) were seen in the first period (from March 2019 to February 2020) and 1012 (51.0%) in the second period (from March 2020 to February 2021).

The age of these patients ranged from 5 to 97 years, with a mean of 47.77 years (with a standard deviation of 13.44 years and a median of 47.21 years). A total of 1,126 (56.9%) were male.

During these two years, 57 patients (2.9%) developed adhesive capsulitis.

Table 1 shows the comorbidities evaluated, as well as their prevalence compared to the total number of patients.

**Table 1:** Comorbidities and their prevalence in relation to patients.

Disease	n	%
SAH	154	6.1
DM	85	3.4
DLP	53	2.1
Hypothyroidism	34	1.3
Hyperthyroidism	4	0.2
Depression	27	1.1
Anxiety	31	1.2

SAH: systemic arterial hypertension; DM: diabetes mellitus; DLP: dyslipidemia.

Table 2 shows the comparison between the two periods regarding age, gender, development of adhesive capsulitis, and comorbidities studied.

**Table 2:** Comparison of the two periods (1st period: from March 2019 to February 2020; 2nd period: from March 2020 to February 2021).

	Period		
Variable	First	Second	p
Age (in years)	47.75 ± 13.49	47.88 ± 13.40	0.708^a^
Male	564 (58.3%)	562 (55.5%)	0.220^b^
SAH	53 (5.5%)	77 (7.6%)	0.053^b^
DM	31 (3.2%)	45 (4.5%)	0.146^b^
DLP	23 (2.4%)	28 (2.8%)	0.576^b^
Hypothyroidism	11 (1.1%)	20 (2.0%)	0.130^b^
Hyperthyroidism	2 (0.2%)	1 (0.1%)	0.617^c^
Depression	6 (0.6%)	18 (1.8%)	0.018^b^
Anxiety	5 (0.5%)	22 (2.2%)	0.001^b^
Capsulitis	16 (1.7%)	41 (4.1%)	0.001^b^

SAH: systemic arterial hypertension; DM: diabetes mellitus; DLP: dyslipidemia; ^a^ descriptive level of Student’s *t*-test probability; ^b^ descriptive level of probability of the chi-square test; ^c^ descriptive level of probability of Fisher’s exact test.

Table 3 shows the comparison of patients with and without adhesive capsulitis in relation to age, gender, and presence of comorbidities studied.

**Table 3:** Comparison of groups of patients with and without adhesive capsulitis.

	Capsulitis		
Variable	No	Yes	p
Age (in years)	46.90 ± 13.65	52.43 ± 9.82	< 0.001^a^
Male	1,435 (58.1%)	29 (50.9%)	0.275^b^
SAH	148 (6.0%)	6 (10.5%)	0.156^c^
DM	77 (3.1%)	8 (14.0%)	< 0.001^c^
DLP	49 (2.0%)	4 (7.0%)	0.030^c^
Hypothyroidism	30 (1.2%)	4 (7.0%)	0.007^c^
Hyperthyroidism	3 (0.1%)	1 (1.8%)	0.087^c^
Depression	22 (0.9%)	5 (8.8%)	< 0.001^c^
Anxiety	23 (0.9%)	8 (14.0%)	< 0.001^c^

SAH: systemic arterial hypertension; DM: diabetes mellitus; DLP: dyslipidemia; ^a^ descriptive level of Student’s *t*-test probability; ^b^ descriptive level of probability of the chi-square test; ^c^ descriptive level of probability of Fisher’s exact test.

## DISCUSSION

Adhesive capsulitis is a pathology with a predominance in females aged between 40 to 60 years old.^1^ ^),(^ ^4^ ^),(^ ^5^

In our study, surprisingly, the number of reported cases was slightly higher in males (50.9%). The mean age of the patients was 52.43 years, within the range normally affected, according to the literature.

We observed that in the second period analyzed, there was a significantly higher percentage of patients with anxiety (p < 0.01) and depression (p = 0.018) who sought the Shoulder Outpatient Clinic, as well as a significant increase in new cases of adhesive capsulitis, whose incidence increased from 1.7% in the pre-pandemic period to 4.1% in the post-pandemic period. This means that the incidence of the disease was 2.41 times higher in the second period (p < 0.001).

Comparing patients who did or did not develop adhesive capsulitis, we observed a significantly higher prevalence of diabetes mellitus, hypothyroidism, anxiety, and depression in the first group.

The prevalence of diabetes mellitus was four times higher in patients with frozen shoulder compared to other patients, approaching the data we have available in the literature, which show that the prevalence of adhesive capsulitis is five to nine times higher in patients with such endocrinopathy.^7^ ^),(^ ^8^

We evaluated the prevalence of hypothyroidism in our sample, whose patients had a five times greater chance of developing frozen shoulder (p = 0.011), agreeing with the work of Cohen et al.,^6^ which showed that in the presence of these disorders (especially hypothyroidism and the presence of nodules in the gland), the chance of a patient developing AC may increase 2.69 times.

In our study, patients with depression and anxiety had a significantly increased risk by 8.8 (p < 0.001) and 14 (p < 0.001) times, respectively, of developing the pathology in question, in agreement with other studies.

In an orthopedic center in Iran, Ebrahimzadeh et al.^11^ analyzed patients with frozen shoulder and showed that 77% of patients had depressive symptoms, while 32% had anxiety symptoms. Both were related to increased pain and limb dysfunction when compared with the control group. Ding et al.^1^ found similar results, showing increased pain, decreased range of motion, and a higher incidence of nocturnal pain in these patients.

Bagheri et al.^12^ concluded that the pain, quality of life (as assessed by the patient), and dysfunction secondary to AC were related more to psychological factors than to the physical parameters evaluated, such as age, gender, and education level.

We believe that the association between mental disorders and adhesive capsulitis justifies the significant increase of the latter, evidenced in our study after the beginning of the pandemic, considering the notorious increase in the number of people with symptoms of stress, depression, and anxiety around this period.

Deng et al.^13^ showed, in their study conducted at the beginning of the pandemic, that more than 28% of the respondent’s reported symptoms of anxiety and more than 37% of depression. In total, 53.8% rated these impacts as moderate or severe, and more than 20% of patients with previous psychological disorders reported worsening symptoms.

Furthermore, Fawas and Samaha^14^ evidenced a proportionality relation between the duration of quarantine and the development of different symptoms of post-traumatic stress disorder. Their study, conducted with Lebanese people undergoing lockdown, compared participants’ complaints after two and four weeks from the start of quarantine. Reports of feeling sad when recalling past experiences, distance from other people, and feelings of hyperactivity or increased wakefulness increased by 29.1%, 25.5%, and 37.3% in the second period participants.

The study had some weaknesses such as its retrospective design and the fact that the diagnosis of psychosomatic illnesses was provided by the patient himself. Further studies on the subject will help to corroborate all these findings.

## CONCLUSION

The study showed a significant increase in the incidence of frozen shoulder after the onset of the COVID-19 pandemic, coinciding with the increase and intensification of psychosomatic symptoms in the population caused by sudden lifestyle changes.

## References

[1] Ding H, Tang Y, Xue Y, Yang Z, Li Z, He D (2014). A report on the prevalence of depression and anxiety in patients with frozen shoulder and their relations to disease status. Psychol Health Med.

[2] Neviaser AS, Neviaser RJ (2011). Adhesive capsulitis of the shoulder. J Am Acad Orthop Surg.

[3] Disser NP, De Micheli AJ, Schonk MM, Konnaris MA, Piacentini AN, Edon DL (2020). Musculoskeletal consequences of COVID-19. J Bone Joint Surg Am.

[4] Ramirez J (2019). Adhesive capsulitis: diagnosis and management. Am Fam Physician.

[5] Lynch TS, Edwards SL (2013). Adhesive capsulitis: current concepts in diagnosis and treatment. Curr Orthop Pract.

[6] Cohen C, Tortato S, Silva OBS, Leal MF, Ejnisman B, Faloppa F (2020). Association between frozen shoulder and thyroid diseases: strengthening the evidences. Rev Bras Ortop.

[7] Park HB, Gwark JY, Jung J (2018). What serum lipid abnormalities are associated with adhesive capsulitis accompanied by diabetes?. Clin Orthop Relat Res.

[8] Whelton C, Peach CA (2018). Review of diabetic frozen shoulder. Eur J Orthop Surg Traumatol.

[9] Zuckerman JD, Rokito A (2011). Frozen shoulder: a consensus definition. J Shoulder Elbow Surg.

[10] Rosner B (1986). Fundamentals of biostatistics.

[11] Ebrahimzadeh MH, Moradi A, Bidgoli HF, Zarei B (2019). The relationship between depression or anxiety symptoms and objective and subjective symptoms of patients with frozen shoulder. Int J Prev Med.

[12] Bagheri F, Ebrahimzadeh MH, Moradi A, Bidgoli HF (2016). Factors associated with pain, disability and quality of life in patients suffering from frozen shoulder. Arch Bone Jt Surg.

[13] Deng J, Zhou F, Hou W, Silver Z, Wong CY, Chang O (2021). The prevalence of depression, anxiety, and sleep disturbances in COVID-19 patients: a meta-analysis. Ann N Y Acad Sci.

[14] Fawaz M, Samaha A (2020). COVID-19 quarantine: post-traumatic stress symptomatology among Lebanese citizens. Int J Soc Psychiatry.

